# Cross-sectional study of gender differences in the perception of body image during cancer-induced weight loss: study protocol for the GRACE study (Global Research on Appearance in Cancer)

**DOI:** 10.1136/bmjopen-2026-116342

**Published:** 2026-07-15

**Authors:** Charlotte Goodrose-Flores, Stephanie Bonn, Ylva Trolle Lagerros, Jann Arends, David Blum, Constanza Figueroa Donoso, Heather Frazier, Ssu-An Lin, Eugenia Romboli, Minh Hoang Van, Ngoc Bao Nguyen, Van Nguyen, Pedro Emilio Perez-Cruz, Linda Bjorkhem-Bergman

**Affiliations:** 1 Division of Clinical Geriatrics, Department of Neurobiology, Care Sciences and Society, Karolinska Institute, Stockholm, Sweden; 2 Department of Medicine, Huddinge, Karolinska Institutet, Stockholm, Sweden; 3 Department of Medicine I, Medical Center – University of Freiburg, Faculty of Medicine, University of Freiburg, Freiburg, Germany; 4 Department of Radiation-Oncology, Competence Center Palliative Care, University Hospital Zurich, Zürich, Switzerland; 5 Carrera Nutrición y Dietética, Escuela Ciencias de la Salud, Facultad de Medicina, Pontificia Universidad Católica de Chile, Santiago, Santiago Metropolitan Region, Chile; 6 School of Mathematics, Science, and Engineering, Department of Nutrition, University of the Incarnate Word, San Antonio, Texas, USA; 7 Palliative Medicine, Harbour Hospice, Auckland, New Zealand; 8 Center for Population Health Sciences, Hanoi University of Public Health, Hanoi, Vietnam; 9 Centro para la Prevención y Control del Cáncer (CECAN), Santiago, Chile; 10 Sección Medicina Paliativa, Escuela de Medicina, Pontificia Universidad Católica de Chile, Santiago, Santiago Metropolitan Region, Chile; 11 Palliative Medicine, Stockholms Sjukhem, Stockholm, Sweden

**Keywords:** PALLIATIVE CARE, Adult oncology, NUTRITION & DIETETICS

## Abstract

**Background:**

Unintentional weight loss is prevalent in advanced cancer and maintaining weight is critical to improve clinical outcomes. Body image plays a significant role in how weight loss is perceived. A previous Swedish study showed that men with advanced cancer had a negative perception of cancer-induced weight loss whereas women perceived the weight loss more positively.

**Objectives:**

The aim of the GRACE study (Global Research on Appearance in Cancer) is to study gender differences in the perception of body image during cancer-induced weight loss. Second, we aim to explore cross-country differences in perceptions of body image.

**Methods and analysis:**

This is a study protocol for a multi-centre, cross-sectional study across six countries (Sweden, Chile, New Zealand, Switzerland, the United States and Vietnam) spanning five continents. We aim to recruit 50 women and 50 men with advanced cancer from each country, targeting 600 people in total. Data will be collected through a structured digital questionnaire covering perceived body image measured with the Body Image Scale (BIS), demographics, anthropometrics, weight history, disease-related symptoms and dietary intake. Data collection started in 2025. Patients are enrolled at each site following ethical approval. The primary endpoint is difference in BIS-score between men and women. A difference of 3 BIS points between groups will be considered clinically significant. For statistical analysis, multivariable linear regression will be used, including country as a fixed effect to account for between-country differences. We will also perform secondary, exploratory analyses examining between-country variation in the gender difference in BIS.

**Ethics and dissemination:**

Ethical approval has been obtained from all of the participating countries. Results will be disseminated through peer-reviewed publications and conference presentations.

**Trial registration number:**

ClinicalTrials.gov (NCT07195448).

**STRENGTHS AND LIMITATIONS OF THIS STUDY**

- Inclusion of participants from six countries across five continents enhances the international relevance of the findings.
- Understanding the perception of body image in patients with advanced cancer is underexplored but highly relevant in clinical practice.
- Variations in healthcare systems and study settings may influence data collection and cross-country comparability.
- The cross-sectional design limits assessment of changes over time.
- Data is self-reported and anonymous.

## Introduction

 Unintentional weight loss is a serious complication of advanced cancer, affecting roughly 80% of patients.^1^ The weight loss is often a result of both metabolic disruptions and/or inadequate dietary intake, leading to malnutrition. Malnutrition in cancer, defined by the progressive depletion of muscle and fat mass and driven by catabolism is, in turn, associated with reduced quality of life, diminished functional capacity and increased mortality.^1^,^3^ Cancer tumours also require energy to grow, following metabolic dysregulation that occurs secondary to inflammatory mediators by the tumour. This can lead to an increase in the body’s resting energy expenditure; often more pronounced in individuals with metastatic disease.^4^ Maintaining body weight, regardless of pre-diagnosis body mass index (BMI), is critical in clinical cancer care, as weight loss has been associated with increased mortality.^5^ Thus, nutritional interventions combined with cancer-directed treatments by healthcare providers may improve survival.

Patients with cancer often experience negative body image due to physical changes caused by tumour burden and/or cancer treatments.^6^ Body image refers to one’s perception and attitudes in relation to one’s own body.^7^ It is a multifaceted construct shaped by early life experiences and intertwined with self-identity.^8^ Our previous study, performed in Sweden, showed that men with advanced cancer had a negative perception of cancer-induced weight loss, whereas women were more likely to perceive weight loss from cancer as a positive consequence.^9^ This poses a challenge as it may contribute to worse outcomes for women compared with men, given the negative effects that weight loss brings. The impact of the disease on body image has been extensively studied in cancers that cause noticeable physical changes, such as breast, colorectal and head and neck cancers.^10^,^12^ However, the perception of weight loss, as an isolated factor, and in relation to body image, remains under-explored.

Further, existing research is largely focused on the negative associations between weight loss and body image and may be overlooking potential cultural and gender differences.^13 14^ Culture is defined as the shared values, beliefs and social norms within a specific group of people. It shapes perceptions of body image, such as the varying emphasis different societies place on thinness as an ideal. This dynamic becomes even more complex when intersecting with gender, which further shapes body image ideals.^15^

By recognising and addressing differences across countries and between men and women, clinical practices and guidelines can be tailored in a gender-sensitive manner, ultimately benefiting patients and potentially improving both quality of life and disease outcomes.

The primary aim of the GRACE-study (Global Research on Appearance in Cancer) is to examine gender differences in perception of body image during cancer-induced weight loss. Second, we aim to explore cross-country differences in perceptions of body image.

## Method

The method section is structured in accordance with the Strengthening the Reporting of Observational Studies in Epidemiology (STROBE) checklist.^16^

### Study design

The GRACE study is a multi-centre, cross-sectional study aimed at recruiting men and women diagnosed with advanced cancer from six countries from five continents. All data in the GRACE study is self-reported in a digital or printed questionnaire.

### Participants

**Figure 1:**
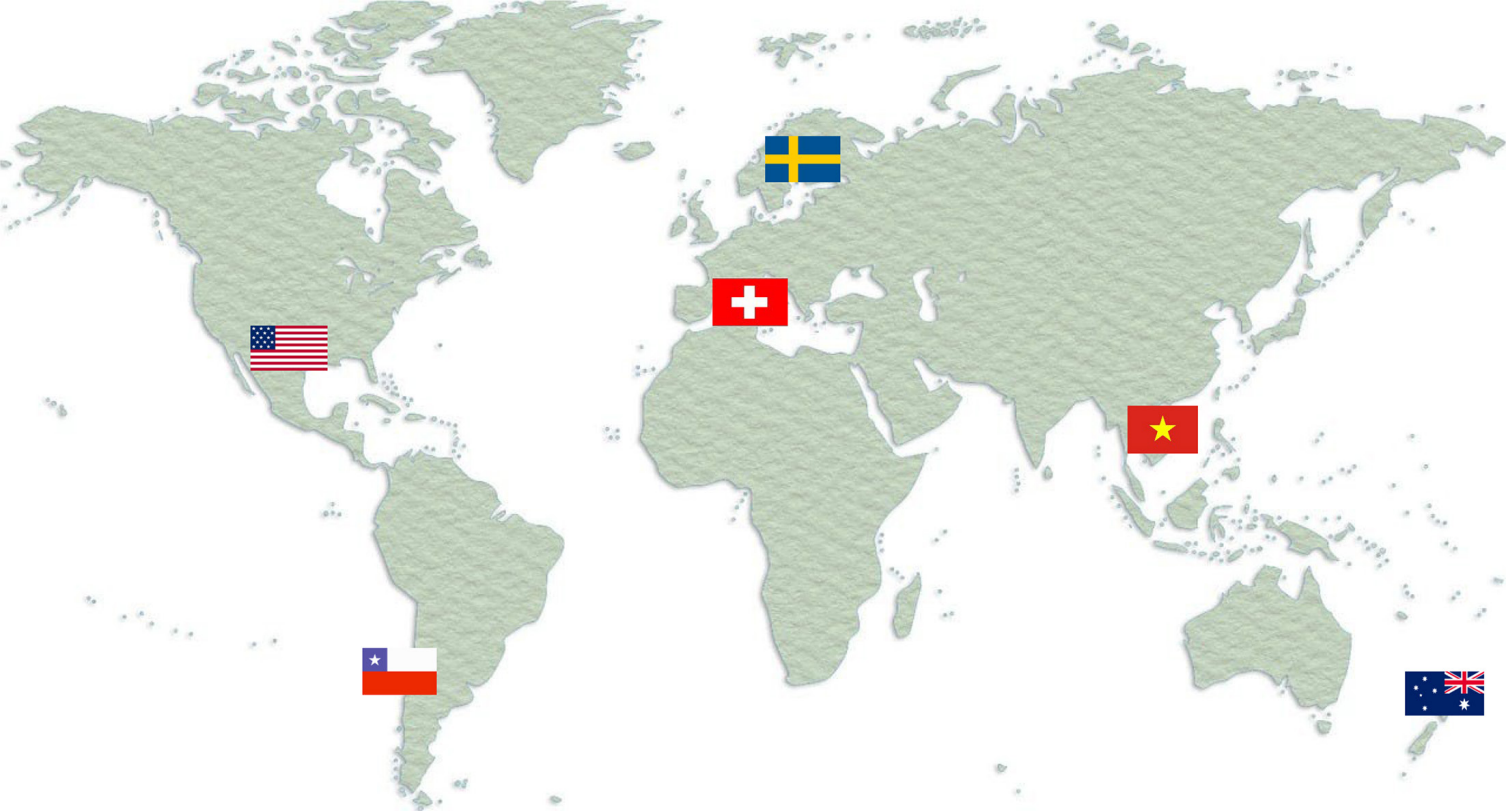
Six countries from five continents are participating in the Global Research on Appearance in Cancer (GRACE) study.

Men and women with advanced cancer, aged 18 years or older will be recruited from oncological clinics or palliative care units in Sweden, Chile, New Zealand, USA, Vietnam and Switzerland.(figure 1)

Different recruitment methods are used by the respective clinical teams in each country. These include posters at the clinic (Switzerland, USA and Vietnam), direct invitation by medical staff (Chile, Sweden, Switzerland, USA and Vietnam), contacting by phone (Chile, New Zealand, Sweden and Vietnam) or by e-mail (New Zealand).

Advanced cancer is defined as incurable cancer in patients with palliative care, needs receiving care in oncological clinics or palliative care units. Eligible patients must be able to complete either a digital or printed version of an anonymous questionnaire with approximately 100 questions.

### Variables

To study perception of body image, all participants will fill in the Body Image Scale (BIS).^17^ BIS includes ten questions and is validated for people with cancer.^17^ One question about sexual attractiveness was excluded as it has been assessed as ‘awkward and embarrassing’ by palliative patients.^17^ Thus, only 9 questions are included in the GRACE study. For each question, there are four possible answer options ranging from ‘not at all’ (0 points) to ‘very much’ (3 points).

In addition, self-reported anthropometric data is collected, including height and current weight, weight prior to diagnosis, and the highest and lowest weight as an adult. Background questions include gender, age, marital status, education, employment status, type of cancer and year of diagnosis. We will also ask the participants to mark the past, current and desired body silhouette (three non-validated silhouettes are presented to each participant). The silhouettes represent BMI categories from <18 to >40 kg/m^2^, although the corresponding BMI values are not disclosed. We will also ask about dietary intake, medications, smoking habits and consumption of alcohol, specified in detail in online supplemental materials (S1).

The validated questionnaire Edmonton Symptom Assessment System (ESAS) will be used to assess disease-related symptoms in the past 24 hours.^18^ The Eastern Cooperative Oncology Group (ECOG) performance scale is used to assess physical functioning.^19^ Finally, self-rated health will be assessed using a visual analogue scale ranging from 0 to 100, with higher scores indicating better perceived health.^20^

All the self-reported questionnaires are presented in online supplemental materials (S1).

The questionnaire was translated from Swedish into American English, New Zealand English, Chilean Spanish, Vietnamese and German with support of native speakers. However, the translated questionnaires have not been validated.

### Country-specific adaptations of the questionnaire

At the end of the original questionnaire, 19 questions have been added to the Chilean version.

These include the Hospital Anxiety and Depression Scale, which evaluates the presence or suspicion of anxiety and depression,^21^ the European Organisation for Research and Treatment of Cancer Quality of Life Questionnaire (EORTC QLQ-C15-PAL)^22^ and the University of California, Los Angeles (UCLA) Loneliness Scale.^23^ In the New Zealand version, the ethnicity question has been changed to a comment box in order to comply with the ethical requirements. Furthermore, in the New Zealand version respondents must confirm that they are 18 years or older before access to the questionnaire.

### Data collection and timeline

All data from the digital questionnaires will be collected and stored in a closed file at Karolinska Institutet, Stockholm, Sweden, where only researchers directly involved in the project have access.

Start of data collection per country: Sweden May 2025, Chile March 2025, New Zealand October 2025, USA May 2025, Vietnam May 2025 and Switzerland June 2026. The estimated end date for completion of data collection in all the participating countries is 31 December 2026.

### Outcome assessments

The primary endpoint is difference in perceived body image during cancer-induced weight loss, measured with BIS, between women and men. The secondary outcome is difference in perceived body image, measured with BIS, between the different countries. As there is no established threshold for a clinically significant difference in BIS, we considered a difference of 3 points to be clinically meaningful based on clinical expertise and previous research.^9^

Missing data will be replaced by the imputed average score of the other questions.^17^ In order to obtain a BIS score, at least 50% of the questions have to be answered.

### Sample size and power calculation

The sample size calculation was based on the primary outcome of body image satisfaction measured using the BIS where a difference of 3 points was considered as a clinically meaningful difference. We assumed an SD of 4.9 (based on the observed variability in our previous study where we had an SD of 3.4 among men and 6.1 among women).^9^ The sample size calculation was based on a two-sided independent samples t-test to detect a difference in mean BIS scores between men and women within a country. Assuming a significance level of 0.05 and 80% statistical power, approximately 45 participants per group are required within a country. To account for potential missing data, the target sample size is set to 50 men and 50 women per country. With six participating countries, the total estimated sample size is 600 participants.

### Statistical analysis

Descriptive data on demographics and other outcomes will be presented as mean values with SD or median values with IQRs for non-normally distributed variables. This will be done for the whole study population and stratified by gender and country. Differences between groups will be evaluated using Student’s t-tests or analysis of variance (ANOVA) to compare means and χ^2^ tests to compare categorical variables. If the continuous data are not normally distributed, non-parametric equivalents will be applied. Missing data will be evaluated with respect to their extent and potential patterns.

The primary analysis will compare BIS between men and women using multivariable linear regression, including country as a fixed effect to account for between-country differences. We will also consider additional variables, such as age, cancer type, cancer stage, BMI and severity of cancer-related weight loss. While these variables are not confounders in a strict causal sense, they represent important prognostic factors and patient characteristics that may differ between sexes and are associated with BIS. We will also perform secondary, exploratory analyses examining between-country variation in the gender difference in BIS.

Additional statistical methods will be applied in accordance with future research questions.

## Discussion

By collecting data from six countries across five continents, the results from this study can be interpreted in an international context and applied to patients in many countries around the world. Attempts were made to include a broader range of countries across both high-income and low-income settings than were ultimately represented.

A patient-centred approach represents an important aspect of clinical care regardless of gender or country. It prioritises outcomes in advanced cancer that matter directly to patients, such as the perception of body image following weight loss, which has been overlooked in previous research.

The findings from the GRACE study will provide a foundation for developing nutrition recommendations tailored to specific countries and genders. Hence, the recommendations may be adapted to the unique needs of each specific population. The use of the same questionnaire, translated into the language spoken in the respective country, across all sites enables comparability and is a strength.

However, variations in healthcare systems and study settings across participating countries may influence data collection procedures, which could, in turn, affect the comparability of cross-country findings. Other limitations include the cross-sectional study design, which limits the ability to capture changes over time, and the questionnaire-based design, which may not fully capture all aspects of body image. Additionally, the data is self-reported and therefore not verified against medical records or other objective measures, which is a limitation. An additional limitation is that the three silhouettes presented to mark past, current and desired body silhouettes have not been validated.

### Significance

Body image perceptions differ between men and women, with women experiencing greater distress and a stronger desire for a slimmer physique compared with men. In advanced cancer, involuntary weight loss is common and often leads to a reduced body size. How such changes in physical appearance are perceived differently across genders remains unexplored. However, maintaining a stable body weight is a key component in advanced cancer care, it may significantly improve patient outcomes. By tailoring interventions depending on individual perspectives on cancer-related weight loss, we can develop targeted protocols which may improve quality of life and prognosis for patients in diverse settings, regardless of gender or country.

### Data availability statement

The raw data will be available from the corresponding author upon reasonable requests.

### Principal investigator

The principal investigator is CGF.

### Ethics and dissemination

Ethical approval has been obtained from the participating countries. Results will be disseminated through peer-reviewed publications and conference presentations.

The digital questionnaire is anonymous, yet respondents will provide consent to participate before responding to any questions. An informed consent statement is presented on the first page of the questionnaire, and participants may only proceed to answer the questions after agreeing to the terms of the study. By accepting these terms, participants provide informed consent prior to inclusion in the study. Data entered by the study participants in the digital questionnaire will be directly stored at secure servers at Karolinska Institutet.

The research will be conducted in accordance with the Declaration of Helsinki. This ensures compliance with both regional and ethical standards and international guidelines for ethical research practice.

Each participating research team has obtained ethical approval from its respective regional review board.

- Sweden, the Swedish Ethical Review Authority, (3.1-2024-002).
- Chile, Health Sciences Ethics Committee from the Pontificia Universidad Católica de Chile, (240723004).
- New Zealand, Auckland Health Research Ethics Committee, (AH 29026).
- Switzerland, (2025-01 605).
- USA, UIW Institutional Review Board, (2025-1618-EX-v4).
- Vietnam, Ethics Committee, University of Public Health, (396/2024/YTCC-HD3).

#### Withdrawal

Invited persons who choose not to respond to the questionnaire do not have to provide a reason. Participants can stop answering the questionnaire at any time. Since the questionnaire is anonymous, it will be impossible to withdraw after submission.

### Patient and public involvement

Patients were involved in the design of this study to ensure that the research procedures and materials were acceptable and appropriate. They were not involved in the conduct, analysis, reporting or dissemination of the research.

## Supplementary material

10.1136/bmjopen-2026-116342 online supplemental file 1
