# Supplementary material for: Cross-sectional study of gender differences in the perception of body image during cancer-induced weight loss: study protocol for the GRACE study (Global Research on Appearance in Cancer)
Source: BMJ Open. 2026 Jul 15;16(7):e116342. doi: 10.1136/bmjopen-2026-116342 (PMC13374447; doi:10.1136/bmjopen-2026-116342)
Supplement: online supplemental file 1 [file bmjopen-16-7-s001.docx]

**Supplemental materials 1 (S1)**

***Data collection***

| *Body image: Body Image Scale (BIS)* (1) measures body image validated for patients with cancer. There are four possible responses between ‘not at all’ (0 points) to ‘very much’ (3 points).^1)^ | |
| --- | --- |
| *Body silhouettes*: Information on participants’ past, current, and desired body frames will be collected.^2)^ Body silhouettes representing BMI categories ranging from <18 to >40 Kg/m^2^, without disclosing the corresponding BMI values in the questionnaire. | |
|  |  |
| ***Background information*** | |
| *Sex* | Female, male, intersex, unspecified |
| *Age* | Years |
| *Type of care* | In-patient, out-patient, advanced medical home care, other |
| *Type of cancer* | Lung, breast, gastrointestinal, genitourinary, hematologic, head-neck, skin, other |
| *Year of diagnosis*  *Current and previous treatment(s)* | Chemotherapy, radiation, endocrine therapy, surgery, immunotherapy, stem cell or bone marrow transplant, other |
| *Additional diagnosis* | Diabetes type 1, diabetes type 2, high blood pressure, high cholesterol, depression, stoma, arthritis, fibromyalgia, allergy, eczema, migraine, Irritable Bowel Syndrome, asthma, kidney disease, eating disorder, other. |
| *Born in another country where receiving cancer treatment* | Yes, no |
| *How many years residing in the country (in years)?* | More than one million inhabitants, between 75.000 and one million inhabitants, less than 75.000 inhabitants. |
| *Employment status* | employed full-time, employed part-time/casual, paid leave, on unpaid leave, sick leave, parental leave, self-employed, not employed, retired, other) |
| *Education* | Did not complete secondary/high school, high school, certificate/diploma, college/university, postgraduate/PhD, other |
| *Ethnicity* | Oceanian, Northwest Europe, Southern and Eastern Europe, North Africa and Middle Eastern, South-East Asian, North-East Asian. Southern and Central Asian, North America, South America, Sub-Saharan Africa, other. |
| *Marital status* | never married, married or domestic partner, divorced/separated/widow/er, other |
| *Children* | Yes/no |
| *If children; how many* | (number) |
|  |  |
| ***Body weight and weight change*** | |
| *Current weight:* | kg (pounds, USA) |
| *Height* | cm (feet and inches, USA) |
| *Weight before the diagnosis* | kg (pounds, USA) |
| *Associate weight loss with the progress of the cancer diagnosis* | Yes, no, don’t know |
| *Look at weight gain as positive from the perspective of the cancer diagnosis:* | Yes, no, don’t know |
| *Would like the weight as before the cancer diagnosis* | Yes, weigh the same, no – more weight, no – less weight, don´t know, other. |
| *Desired weight* | kg (pounds, USA) |
| *Lowest weight as an adult:* | kg (pounds, USA) |
| *Highest weight as an adult:* | kg (pounds, USA) |
| *History of weight loss attempts* | Tried to lose weight 1 to 5 times, tried to lose weight 6 to 10 times |
| *How much weight lost, at the most, during weight loss attempts* | kg (pounds, USA) |
| *Tried to gain weight as an adult before cancer diagnosis* | Never, yes. |
| *Afraid of gaining weight in the past week* | Not at all, a little, quite a bit, very much |
| *Afraid of losing more weight in the past week* | Not at all, a little, quite a bit, very much |
| *Avoided wearing clothes that increase consciousness about the body in the past week* | Not at all, a little, quite a bit, very much |
| *Felt have eaten too much in the past week* | Not at all, a little, quite a bit, very much |
| *Felt that have eaten too little in the past week* | Not at all, a little, quite a bit, very much |
| *Felt dissatisfied about the body when seen in mirror in the past week* | Not at all, a little quite a bit, very much |
| *Felt pressured to eat from family and friends* | Not at all, a little, quite a bit/very much |
| *Experienced a change in eating habits since diagnosis* | Not at all, a little, quite a bit, very much |
| *In what situations did it have an effct* | No effect, when eating out, eating alone at home, eating at home with family/friends, eating at friends’ houses, other |
|  |  |
| ***Nutritional intake*** | |
| *Received dietary recommendations from a dietitian following the cancer diagnosis* | Yes, no |
| *What kind of nutritional education received* | How to modify diet to gain weight, how to modify diet to increase protein intake, modify diet due to diarrhea, modify diet due nausea, modify diet due to changes in taste and smell, prescribed oral nutritional supplements. |
| *Receiving, or received, enteral or parenteral nutrition* | Currently receiving enteral nutrition, currently receiving parenteral nutrition, previously received enteral nutrition, previously received parenteral nutrition, neither enteral nor parenteral nutrition. |
| *Frequency of intake of fruits and/or berries/vegetables, cookies, cakes, chocolate, candy etc. or /soda/fruit juice or smoothie.* | Frequency of intake in the past week: Once per day or more often, 3 to 6 times per week, no intake in the past week) |
| *Hunger* | Time of the day experience the most hunger |
| *Meals* | Consuming meals on a regular schedule, yes/no |
| *Eating* | Would like to eat more, eat less, eat just enough, other |
| *Parenteral/enteral nutrition/oral nutrition supplements in the past week*: | Parenteral nutrition, enteral nutrition, oral nutrition supplements, neither |
| *24-hour food* intake | Free text. Participants are asked to write down all foods and beverages consumed in the previous 24 hours as free text in the questionnaire. Participants are encouraged to include the quantities of foods and beverages consumed, specifying volumes and weights whenever possible. |
| ***Alcohol, tobacco and medications*** | |
| *Medications, alcohol and tobacco use*  *Medications* | Corticosteroids, opioids, medications to decrease blood sugar, antidepressants, neither of the medications mentioned. |
| *Use of nicotine* | Never used nicotine, used to use nicotine, but quit, smoke cigarettes/cigars, use snuff, use e-cigarettes/vapes. |
| *When quit using nicotine* | 0 to 1 year ago, 2 to 5 years ago, 6 or more years ago. |
| *Consumption of alcoholic beverages* | Never, less than weekly, once or twice a week, daily or every other day. |
| *What kind of alcohol* | Wine, beer, cocktails or mixed beverages, cognac, whiskey, gin or other hard liquors |
|  |  |
| ***Symptom burden*** | |
| *Edmonton Symptom Assessment System* (ESAS) assesses disease-related symptoms in the past 24 hours (2) | Participants respond on a scale between 0 (no symptoms) to 10 (severe symptoms) |
| *The ECOG* performance status is a commonly used scale (3) | Participants respond to how well they can carry out daily activities, self-care, work, and how much time they spend resting or confined to bed/chair |
| *Health scale* (4) | Measures self-rated health on a scale between 0 (= the worst health, 100 = the best health) |
| *Experienced constipation in the last week* | Yes, no |
| *Experienced diarrhea in the last week* | Yes, no |
| *Experienced dry mouth in the last week*: | Yes, no |
| *Experienced trouble swallowing in the last week* | Yes, no |
| *Experienced change in taste and smell in the last week* | Yes, no |
| *Experienced early satiety* | Yes, no |
| *Experienced dental problems in the last week* | Yes, no |
| *Receive sufficient support with food shopping and meal preparations* | Yes, no, sometimes, in no need of support, other |

^1)^ One question from the original BIS about sexual attractiveness was excluded as it was assessed as “awkward and embarrassing” to answer. ^2)^The silhouettes have been developed by the team.

**References**

1. Hopwood P, Fletcher I, Lee A, Al Ghazal S. A body image scale for use with cancer patients. European journal of cancer (Oxford, England : 1990). 2001;37(2):189-97.

2. Hui D, Bruera E. The Edmonton Symptom Assessment System 25 Years Later: Past, Present, and Future Developments. Journal of pain and symptom management. 2017;53(3):630-43.

3. Sok M, Zavrl M, Greif B, Srpčič M. Objective assessment of WHO/ECOG performance status. Supportive care in cancer : official journal of the Multinational Association of Supportive Care in Cancer. 2019;27(10):3793-8.

4. Rabin R, de Charro F. EQ-5D: a measure of health status from the EuroQol Group. Annals of medicine. 2001;33(5):337-43.
