# Supplementary material for: Cross-sectional study of gender differences in the perception of body image during cancer-induced weight loss: study protocol for the GRACE study (Global Research on Appearance in Cancer)
Source: BMJ Open. 2026 Jul 15;16(7):e116342. doi: 10.1136/bmjopen-2026-116342 (PMC13374447; doi:10.1136/bmjopen-2026-116342)
Supplement: Supplementary file 2 [file bmjopen-2026-116342.reviewer_comments.pdf]

PEER REVIEW HISTORY

BMJ Open publishes all reviews undertaken for accepted manuscripts. Reviewers are asked to complete a checklist review form and are provided with free text boxes to elaborate on their assessment. These free text comments are reproduced below.

ARTICLE DETAILS

Title (Provisional)

A Cross-Sectional Study of Gender Differences in the Perception of Body Image during Cancer-induced Weight Loss: Study Protocol for the GRACE Study (Global Research on Appearance in Cancer)

Authors

Goodrose-Flores, Charlotte; Bonn, Stephanie; Trolle Lagerros, Ylva; Arends, Jann; Blum, David; Figueroa Donoso, Constanza; Frazier, Heather; Lin, Ssu-An; Romboli, Eugenia; Hoang Van, Minh; Nguyen, Ngoc Bao; Nguyen, Van; Perez-Cruz , Pedro Emilio; Bjorkhem-Bergman, Linda

---

VERSION 1 - REVIEW

Reviewer 1

Name Ishiki, Hiroto

Affiliation National Cancer Center Japan, Department of Palliative Medicine

Date 05-Mar-2026

COI None

The GRACE study protocol presents a well-designed, multi-center cross-sectional study addressing a significant yet under-explored gap in advanced cancer care: the psychological perception of weight loss and body image across different cultures and genders. The study is highly relevant for developing culture-sensitive nutritional interventions.

Reviewer 2

Name Imamura, Yoshinori

Affiliation Kobe University Graduate School of Medicine School of Medicine, Medical Oncology and Hematology

Date 22-Mar-2026

COI None

This international protocol addresses an underexplored and clinically relevant question. However, the manuscript requires substantial clarification of its primary endpoint, estimand, sample size justification, and cross-cultural measurement strategy before the planned analyses can be interpreted with confidence.

#1 Primary endpoint / estimand is unclear.

The manuscript alternates between “perception of weight loss,” “body image,” gender differences, and country differences. Please explicitly define one primary endpoint, one primary comparison or model, and the exact hypothesis being tested.

#2 No prespecified interpretation threshold or effect size.

The protocol should state what magnitude of difference in BIS or other primary measure is considered meaningful, and how the authors plan to interpret sex and country differences.

#3 Sample size justification is insufficient for an international, heterogeneous study.

The current rationale is extrapolated from a prior Swedish study with a much smaller and more homogeneous setting. Please provide a formal sample size calculation based on the actual primary analysis, including assumptions on variance, effect size, and whether interaction terms or country effects will be tested.

#4 Potential confounding and heterogeneity are not adequately addressed.

With equal numbers of men and women per country, important imbalance by cancer type, cancer stager, weight-loss severity, BMI, treatment phase, or prior intentional weight loss may obscure the associations of interest. Please prespecify key covariates and the analytic strategy for adjustment.

#5 Cross-country comparability of the questionnaire is not established.

The manuscript states that the same questionnaire enables comparability, yet country-specific modifications are described. Please clarify translation methodology, whether validated local-language versions exist for each instrument, and how measurement equivalence across languages/countries will be ensured.

#6 Statistical analysis plan is too broad and partly post hoc.

Phrases such as “additional statistical methods will be applied in accordance with future research questions” are too open-ended for a protocol paper. Please specify the primary model, secondary analyses, handling of missing data, multiplicity, and whether country will be treated as a fixed or random effect.

Reviewer 3

Name Kennedy, Fiona

Affiliation University of Leeds, Psychosocial Oncology & Clinical Practice Research Group

Date **31-Mar-2026**

COI **None**

Thank you for submitting this protocol paper reporting a multi-center, cross-sectional study across eight countries. Whilst the paper reports an ongoing study and the authors state that 'data collection started in 2025' there is little information provided about the dates of the study (e.g. specific start/opening dates, anticipated dates of recruitment close, study close etc). Furthermore, the paper would benefit from much more detail and transparency of the study methods (especially recruitment methods, outcomes section and data analysis) as what is reported here is very limited. My suggestions below aim to highlight where further detail is needed.

1. Sample size section - does not actually specify the overall target sample size.

2. The primary outcome is not clearly specified in the outcomes section.

3. How is anthropometrics collected - self reported or from medical records? (including weight prior to diagnosis, highest / lowest weights - how is a participant meant to accurately recall this is self-reported?) Depending on the answer to this, this could be another study limitation.

4. Silhouettes question - is this a validated research tool? If so it should be referenced. How many silhouettes are presented to participants?

5. Please described in detail how participants are recruited to this study (e.g. approached by their clinical team, sent a letter, posters around a hospital/clinic location, or more broad recruitment in communities) - this is important information both for replication and for judging the ethical approach used, and is currently not specified. Furthermore, does the process differ in different countries? If so please briefly specify how it differs.

6. Background questions - why is ethnicity not collected? It is not listed in this section but it is mentioned later in relation to adaptations in New Zealand. Please provide specific detail on how ethnicity question is presented to participants including the response options provided?

7. Statement relating to "respondents must confirm..." - is this just in New Zealand or in all countries?

8. Statement relating to "we will also ask questions about dietary intake, medications, smoking habits and consumption of alcohol..." - this is very vague. How are these questions asked? Using validated measures? More detail is needed to allow for the reader to understand what questions are being asked and what responses are provided or if free text.

9. What question/questionnaire is addressing 'perception of weight loss' (including response options)?

10. Analysis section - discussion of covariate analysis and 'additional statistic methods' is very vague and provides the reader with very little understanding of the analysis plans.

11. Discussion - claims of application to patients globally - can the author really make this claim given that the countries listed are predominantly high income countries with some major omissions e.g. no African countries? Again, i think this point may need to be muted and the lack of some continents/countries being represented be mentioned as a study limitation.

12. Unclear what you mean by 'patient-centred focus' and how this is a strength?

13. Please confirm if the statement regarding all data being stored in Sweden is correct given that much of the data is from other countries - will other countries data also be stored in Sweden?

14. How will advanced cancer be determined? I am uncertain on this as the methods relating to recruitment of participants is poorly described. Please make clear for the reader as per point 5 above.

15. As per above, please provide clear detail on whether all data in this study is self-reported by participants or any from their medical records?

16. Given that this study is recruiting from 8 countries, are you translating the study materials / questionnaires into different languages? If not, this would be another study limitation.

VERSION 1 - AUTHOR RESPONSE

Reviewer: 1
Dr. Hiroto Ishiki, National Cancer Center Japan

Comments to the Author: The GRACE study protocol presents a well-designed, multi-center cross-sectional study addressing a significant yet under-explored gap in advanced cancer care: the psychological perception of weight loss and body image across different cultures and genders. The study is highly relevant for developing culture-sensitive nutritional interventions.

Author response: Thank you for the positive assessment of our study. We greatly appreciate your recognition of the importance of this under-explored area in advanced cancer care and the potential contribution of the study to the field of palliative care.

Reviewer: 2
Dr. Yoshinori Imamura, Kobe University Graduate School of Medicine School of Medicine

Comments to the Author: This international protocol addresses an underexplored and clinically relevant question. However, the manuscript requires substantial clarification of its primary endpoint, estimand, sample size justification, and cross-cultural measurement strategy before the planned analyses can be interpreted with confidence.

#1 Primary endpoint / estimand is unclear.
The manuscript alternates between "perception of weight loss," "body image," gender differences, and country differences. Please explicitly define one primary endpoint, one primary comparison or model, and the exact hypothesis being tested.

Author response: Thank you for highlighting this. We agree that it is important to specify the study aim clearly. We have now re-written the aim of the study to clarify this in the abstract and in the introduction.

Added text in abstract: *Objectives: The aim of the GRACE-study (Global Research of Appearance in Cancer) is to study gender differences in the perception of body image during cancer-induced weight loss. Secondly, we aim to explore cross-country differences in perceptions of body image.*

Added text in Introduction: *The primary aim of the GRACE-study (Global Research on Appearance in Cancer) is to study gender difference in perception of body image, during cancer-induced weight loss. Secondly, we aim to explore cross-country differences in perceptions of body image.*

#2 No prespecified interpretation threshold or effect size.
The protocol should state what magnitude of difference in BIS or other primary measure is considered meaningful, and how the authors plan to interpret sex and country differences.

Author response: Thank you for this suggestion. We have now included a more detailed power calculation and expanded the text in the statistical analysis section. We have considered a difference of 3 points on the Body Image Score (BIS) as a clinically significant difference. Since there is no established threshold for what is considered as a clinical significant difference in BIS we have decided this threshold based on our clinical experience and our previous research using this tool (Goodrose-Flores et al, Life 2022).

Added text: *The sample size calculation was based on the primary outcome of body image satisfaction measured using the body image scale (BIS). As there is no established threshold for a clinically significant difference in BIS, we considered a difference of 3 points to be clinically meaningful based on clinical expertise and previous research. We assumed a standard deviation of 4.9 (based on the observed variability in our previous study where we had an SD of 3.4 among men and 6.1 among women. The sample size calculation was based on a two-sided independent samples t-test to detect a difference in mean BIS scores between men and women within a country. Assuming a significance level of 0.05 and 80% statistical power, approximately 45 participants per group are required within a country. To account for potential missing data, the target sample size is set to 50 men and 50 women per country. With seven participating countries, the total estimated sample size is 700 participants.*

#3 Sample size justification is insufficient for an international, heterogeneous study. The current rationale is extrapolated from a prior Swedish study with a much smaller and more homogeneous setting. Please provide a formal sample size calculation based on the actual primary analysis, including assumptions on variance, effect size, and whether interaction terms or country effects will be tested.

Author response: Thank you for raising this point. We have now included a formal sample size calculation based on the primary outcome of body image perception, measured using the BIS score, and the primary aim of assessing the difference between women and men. The full paragraph for Sample size is outlined in response to comment #2 above.

#4 Potential confounding and heterogeneity are not adequately addressed. With equal numbers of men and women per country, important imbalance by cancer type, cancer stager, weight-loss severity, BMI, treatment phase, or prior intentional weight loss may obscure the associations of interest. Please prespecify key covariates and the analytic strategy for adjustment.

Author response: Thank you for this important comment. We agree that potential confounding and heterogeneity across countries and patient characteristics are critical considerations. We have now clarified the analytical strategy in the Statistical analysis section.

Added text: *The primary analysis will compare BIS between men and women using multivariable linear regression, including country as a fixed effect to account for between-country differences. We will also consider additional variables, such as age, cancer type, cancer stage, body mass index (BMI), and severity of cancer-related weight loss. While these variables are not confounders in a strict causal sense, they represent important prognostic factors and patient characteristics that may differ between sexes and are associated with BIS. We will also perform secondary, exploratory analyses examining between-country variation in the gender difference in BIS.*

#5 Cross-country comparability of the questionnaire is not established. The manuscript states that the same questionnaire enables comparability, yet country-specific modifications are described. Please clarify translation methodology, whether validated local-language versions exist for each instrument, and how measurement equivalence across languages/countries will be ensured.

Author response: Thank you for the constructive comment. The same questionnaire is used in every country and translated into the main language spoken in the respective country. We have now clarified this in “data collection” section. The modifications in a few of the questionnaires were primarily based on the respective ethics’ requirements, such as assuring

the age 18 or above and making the question about ethical background replied in a comment box. This is highlighted in the section “Country-specific adaptations of the questionnaire”.

Added text: *The questionnaire was translated into American English, New Zealand English, Chilean Spanish, German, Swedish and Vietnamese with support of native speakers. The questionnaire has not been validated.*

Added text in discussion: *The use of the same questionnaire, translated into the language spoken in the respective country, across all sites enables comparability and is a strength.*

#6 Statistical analysis plan is too broad and partly post hoc.
Phrases such as “additional statistical methods will be applied in accordance with future research questions” are too open-ended for a protocol paper. Please specify the primary model, secondary analyses, handling of missing data, multiplicity, and whether country will be treated as a fixed or random effect.

Author response: Thank you for highlighting this. In response to previous comments and this, we have now revised the manuscript and clarified the primary aim and outcome in the “outcome assessment” section (see answer #4) and added a more detailed power calculation (see answer #2), and revised the statistical analysis paragraph.

Added text in statistical analysis: *Missing data in variables included in the body image score (BIS) will be replaced by the imputed average score of the other questions, per standard (Hopwood et al, Eur J Cancer 2001). However, in order to obtain a BIS score, at least 50% of the included questions must be answered. Missing data in other variables will be evaluated with respect to their extent and potential patterns. We will address our primary aim by comparing body image satisfaction (BIS) between women and men using multivariable linear regression. Country will be included as a fixed effect to account for differences between countries. We will also consider additional variables, such as age, cancer type, cancer stage, body mass index (BMI), and severity of cancer-related weight loss as these represent important prognostic and case-mix factors that may differ between sexes and are associated with BIS. We will also perform secondary, exploratory analyses examining between-country variation in the sex difference in BIS.*

Reviewer 3:
Dr. Fiona Kennedy, University of Leeds

Comments to the Author:
Thank you for submitting this protocol paper reporting a multi-center, cross-sectional study across eight countries. Whilst the paper reports an ongoing study and the authors state that 'data collection started in 2025' there is little information provided about the dates of the study (e.g. specific start/opening dates, anticipated dates of recruitment close, study close etc). Furthermore, the paper would benefit from much more detail and transparency of the study methods (especially recruitment methods, outcomes section and data analysis) as what

is reported here is very limited. My suggestions below aim to highlight where further detail is needed.

1. Sample size section - does not actually specify the overall target sample size.

Author response: Thank you for your comment. We have now added information about the overall sample size in the Sample size section.

Added text: *The sample size calculation was based on the primary outcome of body image satisfaction measured using the body image scale (BIS). As there is no established threshold for a clinically significant difference in BIS, we considered a difference of 3 points to be clinically meaningful based on clinical expertise and previous research. We assumed a standard deviation of 4.9 (based on the observed variability in our previous study where we had an SD of 3.4 among men and 6.1 among women (Goodrose-Flores et al, Life, 2022). The sample size calculation was based on a two-sided independent samples t-test to detect a difference in mean BIS scores between men and women within a country. Assuming a significance level of 0.05 and 80% statistical power, approximately 45 participants per group are required within a country. To account for potential missing data, the target sample size is set to 50 men and 50 women per country. With seven participating countries, the total estimated sample size is 700 participants.*

2. The primary outcome is not clearly specified in the outcomes section.

Author response: Thank you for highlighting this issue. We have now clearly specified the primary aim of the study in the abstract and in the Aims section of the introduction. We have also added a more detailed description of outcome assessment in the "outcome assessment" section.

Added text in abstract: *Objectives: The aim of the GRACE-study (Global Research of Appearance in Cancer) is to study gender differences in the perception of body image during cancer-induced weight loss. Secondly, we aim to explore cross-country differences in perceptions of body image.*

Added text in Introduction: *The primary aim of the GRACE-study (Global Research on Appearance in Cancer) is to study gender difference in perception of body image, during cancer-induced weight loss. Secondly, we aim to explore cross-country differences in perceptions of body image.*

Added text in Outcome assessment: *The primary endpoint is difference in perceived body image during cancer-induced weight loss, measured with BIS, between women and men. The secondary outcome is difference in perceived body image, measured with BIS, between the different countries. As there is no established threshold for a clinically significant difference in BIS, we considered a difference of 3 points to be clinically meaningful based on clinical expertise and previous research (Goodrose-Flores et al, Life, 2022).*

Missing data will be replaced by the imputed average score of the other questions (Hopewood, Eur J Cancer 2001. In order to obtain a BIS score, at least 50% of the questions have to be answered.

3. How is anthropometrics collected - self reported or from medical records? (including weight prior to diagnosis, highest / lowest weights - how is a participant meant to accurately recall this is self-reported?) Depending on the answer to this, this could be another study limitation.

Author response: This is an important issue. Thank you. In this study anthropometric data is self-reported, as are all data collected via a questionnaire. In the Study Setting section, we have now clarified that the data were self-reported to avoid any ambiguity. All questions included in the questionnaire are outlined in the new "Supplementary material", which hopefully, also makes it clearer that this is self-reported data. We have also included the self-reported questionnaire as a limitation in the Discussion section.

Added text in the methods, study design section: *All data in the GRACE study is self-reported in digital questionnaire.*

Added text in the methods, variables section: *All the self-reported questionnaire are presented in Supplementary materials (S1).*

Added text in the discussion: *Additionally, the data is self-reported and therefore not verified against medical records or other objective measures, which is a limitation.*

4. Silhouettes question - is this a validated research tool? If so it should be referenced. How many silhouettes are presented to participants?

Author response: Thank you for requesting clarification regarding the silhouettes. There are three silhouettes, it is not a validated research tool. We have now added that the instrument is not validated in the Outcome assessments section and in the Discussion section as a limitation

Added text in the data collection section: *We will also ask the participants to mark the past, current, and desired body silhouette (three non-validated silhouettes are presented to each participant). The silhouettes represent BMI categories from <18 to >40 kg/m2, although the corresponding BMI values are not disclosed.*

Added text in the discussion: *An additional limitation is that the three silhouettes presented to mark past, current, and desired body silhouettes have not been validated.*

5. Please describe in detail how participants are recruited to this study (e.g. approached by their clinical team, sent a letter, posters around a hospital/clinic location, or more broad recruitment in communities) - this is important information both for replication and for judging the ethical approach used, and is currently not specified. Furthermore, does the process differ in different countries? If so please briefly specify how it differs.

Author response: This is an important point to explain. Thank you for highlighting this. We have now added this in the section “participants” in the methods.

Added text: *Men and women with advanced cancer, aged 18 years or older will be recruited from oncological clinics or palliative care units in Sweden, Chile, New Zealand, USA, Vietnam, Switzerland and Germany. Different recruitment methods are used in the clinical team in each respective country. These include posters at the clinic (Germany, Switzerland, United States, Vietnam), direct invitation by medical staff (Chile, Germany, Sweden, Switzerland, United States, Vietnam), contacting by phone (Chile, New Zealand, Sweden, Vietnam) or by e-mail (New Zealand).*

6. Background questions - why is ethnicity not collected? It is not listed in this section but it is mentioned later in relation to adaptations in New Zealand. Please provide specific detail on how ethnicity question is presented to participants including the response options provided?

Author response: Thank you for the comment and for giving us the opportunity to clarify. Information on ethnicity is collected (see Supplementary material for all questions included in the study questionnaire). However, as we were developing the questionnaire, it became apparent that the question about ethnicity was considered sensitive to ask in certain cultures. Furthermore, the question was not approved by the ethical board in New Zealand. Therefore, the response options were revised to reflect the cultural considerations and ethical requirements of each country.

7. Statement relating to "respondents must confirm..." - is this just in New Zealand or in all countries?

Author response: Thank you for your comment. We have now clarified and added New Zealand as the country with the requirement to assure the respondent to be 18 years of age or older. This has been added in the section for Country-specific adaptations of the questionnaire.

Added text: *Furthermore, in the New Zealand version respondents must confirm that they are 18 years or older before access to the questionnaire.*

8. Statement relating to "we will also ask questions about dietary intake, medications, smoking habits and consumption of alcohol..." - this is very vague. How are these questions asked? Using validated measures? More detail is needed to allow for the reader to understand what questions are being asked and what responses are provided or if free text.

Author response: We have now including a supplementary file where all questions are outlined in detail (S1). We have also clarified in the methods that we aske about dietary intake, medications, smoking habits, and alcohol consumption.

Added text: *We will also ask about dietary intake, medications, smoking habits and consumption of alcohol, specified in detail in supplementary materials (S1)*

9. What question/questionnaire is addressing 'perception of weight loss' (including response options)?

Author response: Thank you for pointing this out. We have now rephrased this wording to "perception of body image" throughout the manuscript. Perception of body image is addressed through the validated Body Image Scale (BIS) questionnaire.

Added text in the methods: *To study perception of body image, all participants will fill in the Body Image Scale (BIS) (17). BIS includes nine questions and is validated for people with cancer (17). One question about sexual attractiveness was excluded as it has been assessed as "awkward and embarrassing" by palliative patients (17). Thus, only 8 questions are included in the GRACE study. For each question, there are four possible answer options between "not at all" (0 points) to "very much" (3 points).*

10. Analysis section - discussion of covariate analysis and 'additional statistic methods' is very vague and provides the reader with very little understanding of the analysis plans.

Author response: We agree that this is an important aspect that needs to be better addressed. We have now added clarifications in the Statistical analysis section.

Added text in outcome assessment: *The primary endpoint is difference in perceived body image during cancer-induced weight loss, measured with BIS, between women and men. The secondary outcome is difference in perceived body image, measured with BIS, between the different countries. As there is no established threshold for a clinically significant difference in BIS, we considered a difference of 3 points to be clinically meaningful based on clinical expertise and previous research (9). Missing data will be replaced by the imputed average score of the other questions (17). In order to obtain a BIS score, at least 50% of the questions have to be answered.*

Added text in statistical analysis: *The primary analysis will compare BIS between men and women using multivariable linear regression, including country as a fixed effect to account for between-country differences. We will also consider additional variables, such as age, cancer type, cancer stage, body mass index (BMI), and severity of cancer-related weight loss. While these variables are not confounders in a strict causal sense, they represent important prognostic factors and patient characteristics that may differ between sexes and are associated with BIS. We will also perform secondary, exploratory analyses examining between-country variation in the gender difference in BIS.*

11. Discussion - claims of application to patients globally - can the author really make this claim given that the countries listed are predominantly high income countries with some major omissions e.g. no African countries? Again, i think this point may need to be muted and the lack of some continents/countries being represented be mentioned as a study limitation.

Author response: Thank you for this important comment. We have now rephrased the sentence in the discussion about the claim about global reach to soften the wording. We have also added information stating that attempts were made to reach a broader range of countries across additional continents. Unfortunately, it was not possible to establish fruitful contact. This has been added to the Discussion section,

Added text: *By collecting data from seven countries across four continents, the results from this study can be interpreted in an international context and applied to patients in many countries around the world. Attempts were made to include a broader range of countries across both high- and low-income settings than were ultimately represented.*

12. Unclear what you mean by 'patient-centred focus' and how this is a strength?

Author response: Thank you for highlighting this unclarity. We have now rephrased the sentence in the Discussion section to clarify its meaning.

Added text: *A patient-centered approach represents an important aspect of clinical care regardless of gender or country. It prioritizes outcomes in advanced cancer that matter directly to patients, such as the perception of body image following weight loss, which has been overlooked in previous research.*

13. Please confirm if the statement regarding all data being stored in Sweden is correct given that much of the data is from other countries - will other countries data also be stored in Sweden?

Author response: This is a very important point and we thank you for raising this question. Yes, all data will only be stored at Swedish servers. Data is collected using digital questionnaires, which are created by first author CGF in each respective language. All questionnaires are created within the same system, accessed through Karolinska Institutet. A QR-code is emailed to each research team for further distribution of the country specific questionnaire to potential study participants. Participants responses to the digital questionnaire are directly stored at servers at Karolinska Institutet, Sweden. We have now clarified this in the manuscript and information about the storage of the digital data is provided in the Data Availability Statement and the Ethics Declaration

Added text: *All data from the digital questionnaires will be collected and stored in a closed file at Karolinska Institutet, Stockholm, Sweden, where only researchers directly involved in the project have access.*

14. How will advanced cancer be determined? I am uncertain on this as the methods relating to recruitment of participants is poorly described. Please make clear for the reader as per point 5 above.

Author response: Thanks for the opportunity to clarify this definition in the manuscript. We have now defined advanced cancer in the Patients/material and methods. Furthermore, the recruiting process has been explained under #5.

Added text: *Advanced cancer is defined as incurable cancer in patients with palliative care needs receiving care in oncological clinics or palliative care units.*

15. As per above, please provide clear detail on whether all data in this study is self-reported by participants or any from their medical records?

Author response: Thank you for this comment. In the "study setting" section we have clarified that all data are self-reported. All questions included in the questionnaire are outlined in the new Supplementary file 1.

Added text: *All data in the GRACE study is self-reported in digital questionnaire.*

Added text in the discussion: *Additionally, the data is self-reported and therefore not verified against medical records or other objective measures, which is a limitation.*

16. Given that this study is recruiting from 8 countries, are you translating the study materials / questionnaires into different languages? If not, this would be another study limitation.

Author response: Thank you for this important reflection. We have now clarified in the methods section that the questionnaire was translated into American English, New Zealand English, Chilean Spanish, German, Swedish and Vietnamese with support of native speakers. We have now also clarified in the Discussion section that the questionnaire has been translated into the language spoken in respective countries.

Added text: *The questionnaire was translated from Swedish into American English, New Zealand English, Chilean Spanish, German and Vietnamese with support of native speakers. However, the translated questionnaire has not been validated.*

Added text: *The use of the same questionnaire, translated into the language spoken in the respective country, across all sites enables comparability and is a strength.*

VERSION 2 - REVIEW

**Reviewer**	**3**
**Name**	**Kennedy, Fiona**

Affiliation **University of Leeds, Psychosocial Oncology & Clinical Practice Research Group**

Date **02-Jun-2026**

COI

Thank you for revising this paper in line with my previous comments (and those of the other reviewers and editor). I am happy with the revisions made and have no further comments.

VERSION 2 - AUTHOR RESPONSE

Reviewer: 3
Dr. Fiona Kennedy, University of Leeds

Comments to the Author:
Thank you for revising this paper in line with my previous comments (and those of the other reviewers and editor). I am happy with the revisions made and have no further comments.

Thank you for confirming that you are satisfied with the revision.

Reviewer: 3
If you have selected 'Yes' above, please provide details of any competing interests.: Not applicable.

This comment is included in the originally submitted version:

"Disclosure statements

The authors report there are no competing interests to declare."
